# Efficacy of a dietary supplement derived from five edible plants on telomere length in Thai adults: A randomized, double‐blind, placebo‐controlled trial

**DOI:** 10.1002/fsn3.3851

**Published:** 2023-11-20

**Authors:** Kemika Praengam, Siriporn Tuntipopipat, Chawanphat Muangnoi, Chatdao Jangwangkorn, Olan Piamkulvanich

**Affiliations:** ^1^ Institute of Nutrition Mahidol University Nakhon Pathom Thailand; ^2^ Research Physician Bangkok Thailand; ^3^ Wincell Research Laboratory Samut Prakan Thailand

**Keywords:** antiaging, five edible plants, telomeres, total antioxidant capacity

## Abstract

Mylife/Mylife100® is a dietary supplement consisting of black sesame seed, guava fruit, mangosteen aril, pennywort leaves, and soy protein. These edible plants contain multiple high‐potential bioactive compounds exerting various vital biological functions including antioxidants which contribute to delaying the rate of telomere shortening. Telomere length is associated with cellular aging and age‐related diseases. This study aimed to assess the efficacy of Mylife/Mylife100® on telomere length through a randomized, double‐blind placebo‐controlled trial. The trial assessed the alteration of leukocyte telomere length after 32 adults aged 50–65 years received either Mylife/Mylife100® or placebo (five capsules/day) for 8‐week supplementation. The results demonstrated a significant increase in mean telomere length from baseline (6313 bp) to the 8‐week supplementation period (6655 bp; *p* < 0.05) in the group receiving the product, whereas no significant change was observed in the placebo group. Additionally, the product group exhibited a significant improvement in plasma total antioxidant capacity levels compared to the placebo group (mean change, +35 vs −38; *p* = 0.006). This study also showed a significant correlation between telomere length and % CD4 + T cells (*r* = +0.325; *p* = 0.00003), % CD8 + T cells (*r* = +0.156; *p* = 0.048), and visceral fat (*r* = − 0.349; *p* = 0.000006). The findings suggest that consuming this dietary supplement (Mylife/Mylife100®) for 8 weeks has a positive effect on cellular aging by lengthening telomeres possible through their antioxidant capacities. Oxidative stress and cellular aging are underlying predisease mechanisms that might be alleviated by supplementing with this product.

## Graphical abstract

The research suggests that consuming a dietary supplement (Mylife/Mylife100®), which includes black sesame seeds, guava fruit, mangosteen aril, pennywort leaves, and soy protein, for 8 weeks has a positive impact on cellular aging by lengthening telomeres possible through their antioxidant capacities.

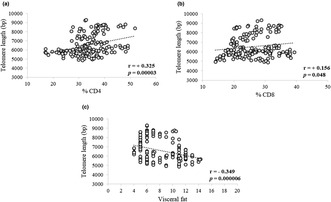

## 1 INTRODUCTION

Telomeres are protein–DNA complexes localized at the ends of linear chromosomes. They are constituted by (TTAGGG)n sequences and associated proteins that protect the end of the chromosome from improper recombination and fusion with another telomere or DNA end (Blackburn, 2001). Telomerase is a vital enzyme for telomere formation, maintenance, and restoration (Blackburn, 2005; Epel et al., 2004). Telomere lengths shorten when cells undergo proliferation, and their length is inversely correlated with age (Monaghan & Ozanne, 2018). Various factors, such as poor health behaviors, high stress, smoking, and low socioeconomic status, obesity, age‐related chronic diseases, and aging, are associated with shortened telomere length (Bekaert et al., 2005; Epel et al., 2004; Valdes et al., 2005; von Zglinicki & Martin‐Ruiz, 2005). These factors result in increased cellular oxidative stress, which is a major contributor to telomere shortening by inducing DNA damage from oxidized bases (Mastalerz‐Migas et al., 2006) or single‐ or/and double‐strand breaks (Passos et al., 2007). Immune cells are unique somatic cells that can upregulate telomerase and limit telomere attrition during cell proliferation after antigenic activation (Kaszubowska, 2008). The adaptive immunity declines with age due to a progressive decline in naive T and B cells (Scholz et al., 2013) and an increase in incompetent memory T cells (Weng, 2008). Previous studies found that memory CD4+ and CD8+ T cells had shorter telomere lengths than naive CD4+ and CD8+ T lymphocytes (Rufer et al., 1999; Weng et al., 1995). A previous study found that telomere length had a positive correlation with B‐ and CD8+ T‐cell responses against influenza vaccine/antigen in elderlies (Najarro et al., 2015). The combined effects of oxidative stress, inflammation, and repeated cell replication contribute to the association between telomere length and chronological aging as well as age‐related disease status. Although telomere shortening cannot be avoided during aging, telomeres can be maintained or even lengthened (Aviv et al., 2009; Ehrlenbach et al., 2009; Epel et al., 2008; Farzaneh‐Far et al., 2010; Nordfjäll et al., 2009).

Mylife/Mylife100® was registered as a dietary supplement by Thai FDA. It consists of water extract powder from pennywort (*Centella asiatica*) leaves, black sesame seed (*Sesamum indicum*), soybean seed (*Glycine max*), guava (*Psidium guajava*) fruit, and mangosteen (*Garcinia mangostana*) aril. Pennywort leaves have been demonstrated to activate telomerase activity in human leukocytes (Tsoukalas et al., 2019). A bioactive extract from pennywort leaves prevented telomere shortening and repressing the decrease of telomerase protein expression compared to the controls in the HEK293 cell cultures. Administration of this bioactive extract increased survival rate of *D. melanogaster* (Karsono et al., 2021). The leaf extract improved cognitive functions through increasing antioxidant gene expression in mouse brain (Gray et al., 2016). Soybean‐derived bioactive peptides possess antioxidant, immunoregulatory, and anti‐inflammatory activities (Kim et al., 2021). Guava pulp and peel reduced lipid peroxidation in hypercholesterolemic rats (Maryanto, 2013). Isoprenylated xanthones in mangosteen demonstrated antioxidant, antinociceptive, and anti‐inflammatory effects (Ovalle‐Magallanes et al., 2017). Black sesame seeds possess strong antioxidant and anti‐inflammatory effects (Yang et al., 2018). Lignans in sesame seeds showed antiaging effects (Jang et al., 2019).

Regular consumption of a diet rich in antioxidant activity and/or diet containing telomerase activators may be able to delay telomere attrition or lengthen telomere length. Various phytochemicals in dietary plants have been reported to exert strong antioxidant effects (Jideani et al., 2021; Nwozo et al., 2023; Zhang et al., 2015). In addition, a recent review reported the role of polyphenolic foods in longevity through their multiple functional properties, including enhancing telomerase activity and stabilizing telomere length (Meccariello & D'Angelo, 2021). Due to the high potential bioactive compounds in the five edible plants of Mylife/Mylife100®, we hypothesized that consuming a certain amount of these plants together (Mylife/Mylife100®), within a certain period of time may prevent or delay telomere attrition and/or lengthen telomere length through their antioxidant and/or activation of telomerase. Therefore, telomere length from peripheral blood mononuclear cell (PBMC) was preliminarily collected from 10 healthy voluntary consumers (two males and eight females; age 40–65 years) by researchers of the selling company. Data demonstrated that telomere lengths were increased by 408 ± 577.4 (mean ± SD) base pairs after the consumers took five capsules/day of this product for 8 weeks without observed adverse effects (monitored from levels of liver enzymes, blood urea nitrogen, and creatinine). Due to these promising data on the increment of telomere length, a randomized, double‐blind, placebo‐controlled trial was conducted in the present study for assessing the effect of this dietary supplement, namely, Mylife/Mylife100® on telomere length from PBMC of Thai adults. Additionally, total leukocyte count, T‐lymphocyte subsets, plasma antioxidant capacity, and basic blood biochemistry were also evaluated.

## 2 MATERIALS AND METHODS

### 2.1 Preparation of product from five edible plants

Dietary supplement product Mylife/Mylife100® was prepared from five edible plants. Briefly, mangosteen aril juice powder was prepared by mixing aril mangosteen with water and crushing it with a blender to obtain slurry. The slurry was then finely ground using a colloid mill to achieve homogeneity. The mangosteen juice was obtained by centrifuging and filtering through a decanter at 1400 rpm to separate the juice from the residue. The juice was heated at 70°C for 30 min using a stream boiler and then dried by a spray dryer (inlet temperature 250°C, outlet temperature 90°C) to produce mangosteen aril juice powder. Pennywort leaf powder was prepared by mixing dry pennywort leaves with water, heating them at 70°C for 30 min by a stream boiler, and filtering the mixture through a decanter by centrifuging at 1400 rpm to obtain a water extract. The water extract was dried by a spray dryer to produce pennywort leaf powder. Guava fruit juice powder was prepared by cutting guava fruits into small pieces, mixing them with water, and grinding them by a juice pressing machine to separate the pulp from the juice. The juice was then filtered through a decanter by centrifuging at 1400 rpm. Subsequently, the juice was heated at 70°C for 30 min by a stream boiler and dried by a spray dryer to produce guava fruit juice powder. Black sesame seed powder was prepared by mixing black sesame seeds with water, grinding them, and filtering the mixture through a decanter by centrifuging at 1400 rpm to obtain sesame milk. The sesame milk was heated at 70°C for 30 min by a stream boiler and dried by a spray dryer to produce black sesame seed powder. Isolated soybean protein was prepared by mixing soybeans with water and grinding them by a juice pressing machine to separate soybean milk from the soybean meal. The soybean milk was centrifuged at 1400 rpm and filtered through a decanter to remove any remaining residue. Subsequently, the soybean milk was heated at 70°C for 30 min by a stream boiler and dried by a spray dryer to produce isolated soy protein powder. The moisture content of all powders was 3%–5%. Each capsule of the supplement contains a combination of 150‐mg pennywort leaf powder (from fresh weight (FW) 750 mg), 100‐mg black sesame seed powder (from FW 2 g), 100‐mg isolated soy protein powder (from FW 400 mg), 100‐mg guava fruit juice powder (from FW 2 g), and 50‐mg mangosteen aril juice powder (from FW 250 mg) as active ingredients. The final product was registered in the Thai FDA as a dietary supplement.

### 2.2 Study participants

Thai adults were recruited through direct contact or poster advertisements around Salaya Campus of Mahidol University, Thailand. A total of 60 participants were enrolled for screening, only 32 participants completed the study. Initial screening was conducted through online and telephone interviews to assess health history, medication use, dietary supplement intake, COVID‐19 vaccination, and other health behaviors. Inclusion criteria for the study were as follows: Thai male or female participants aged 50–65 years with a BMI of 20–24.9 kg/m^2^, systolic blood pressure (SBP) ≤ 140 mmHg, diastolic blood pressure (DBP) ≤ 90 mmHg, nonsmokers, nonalcohol drinkers, blood urea nitrogen levels between 7.8 and 20.3 mg/dL, creatinine levels of 0.81–1.43 mg/dL for males and 0.65–1.08 mg/dL for females, aspartate transaminase (AST) levels of 0–35 U/L for males and 0–31 U/L for females, alanine transaminase (ALT) levels of 0–45 U/L for males and 0–34 U/L for females, and estimated glomerular filtration rate greater than 60 mL/min/1.73 m^2^. Participants on a fixed dose of either blood pressure medications and/or lipid‐lowering medications without serious symptoms were also recruited. Female participants had to have gone through menopause for at least 6 months. The recruited participants had not taken any dietary supplements and herbal supplements for at least 2 months before starting the trial. Participants had to refrain from consuming pennywort leaves, mangosteen fruit, guava fruit, black sesame seed, and soybean or their products for at least 2 weeks before starting and during the trial. Exclusion criteria included type 2 diabetes, cancer, renal and/or hepatic disease, thyroid disorder, pregnant women, lactating women, and participants who were immunized with any vaccine in the past month.

### 2.3 Study design and protocol

This is a randomized, double‐blind, placebo‐controlled, parallel‐group study. Each participant received only placebo or supplement capsules (Mylife/Mylife100®) for 8 weeks. The principal investigator, participants, and laboratory analysts were blinded until the study was completed. This study was conducted following the guidelines of the Declaration of Helsinki, and all procedures involving human participants were approved by the Central Institutional Review Board of Mahidol University under protocol number MU‐CIRB2021/409.1409. Written informed consent was obtained from all participants prior to conducting the study. The study was registered at clinicaltrials.in.th under the registration number TCTR20230222009.

The study spanned over 16 weeks. Each participant had five visits scheduled during the study period, at baseline or week 0 (the first visit), 4 weeks (second visit), 8 weeks (third visit), 12 weeks (fourth visit), and 16 weeks (fifth visit). Body composition data and blood samples were collected every visit. Participants were asked to maintain their lifestyle, including their daily dietary intake and exercise habits as usual as possible during the entire 16‐week period. Their dietary intakes were recorded for 3 days/week, including 2 weekdays and 1 weekend day, for the entire 16 weeks. During the initial 8‐week period, participants maintained their usual free‐living lifestyle without taking any supplements. Before starting the following 8‐week supplementation period, the participants were randomly assigned to receive either the placebo or the supplements by a random number table. At the third visit, participants were given a 4‐week supply of capsules to consume at home, and they were provided with another 4‐week supply of capsules at the fourth visit. The participants were asked to record their daily consumption of capsules and report any adverse effects. If they experienced any discomfort after taking the capsules, they could immediately consult medical doctor of the research project. Researchers used application line to set up line groups for recalling capsule consumption daily. If they forgot to consume the capsule in the morning, the capsules were consumed before lunch. If they forgot to consume in the evening, they were taken before bedtime. The number of capsules from the previous visit was counted to check for compliance. Participants who consumed less than 90% of the total capsules were excluded from the analysis. Initially, 18 participants received five capsules/day of placebo, while the other 19 participants received five capsules/day of the product during the supplementation period. Two capsules were taken in the morning and three capsules were taken in the evening on an empty stomach. Both the product and placebo capsules were provided by Asian Phytoceuticals Public Company Limited (Bangkok, Thailand). A placebo capsule contained 500 mg of corn starch.

### 2.4 Blood sample collection

Blood samples were collected by venipuncture following a 12‐h fasting period to measure several biomarkers. Serum was collected from clot blood. Plasma was separated from the blood samples and stored at −80°C for measurement of total antioxidant capacity. An aliquot of EDTA blood was used to perform a complete blood count and measure T‐lymphocyte subpopulation. Another EDTA blood sample was used for the isolation of peripheral blood mononuclear cells (PBMCs) to measure absolute telomere length.

### 2.5 Measurement of blood biochemistry and body composition

Plasma glucose, serum total cholesterol, high‐density lipoprotein cholesterol (HDL‐c), triglycerides (TG), aspartate transaminase (AST), alanine transaminase (ALT), uric acid, blood urea nitrogen (BUN), and creatinine were analyzed using specific assay kits in an automatic analyzer. Body weight, height, body mass index (BMI: kg/m^2^), body fat (% of body weight), fat mass (kg), fat‐free mass (FFM: kg), muscle mass (kg), total body water (%), bone mass (kg), and visceral fat were measured using a Tanita BC‐420MA segmental body composition analyzer (Tanita Corporation, Tokyo, Japan).

### 2.6 Measurement of plasma total antioxidant capacity

Ferric reducing antioxidant power (FRAP) assay was used to measure the plasma total antioxidant capacity (TAC). Briefly, the FRAP reagent was freshly prepared by mixing 300‐mM acetate buffer (pH 3.6), 10‐mM TPTZ (2,4,6‐tripyridyl‐s‐triazine) solution in 40‐mM hydrochloric acid, and 20‐mM iron (III) chloride at a ratio of 10:1:1. To conduct the assay, 150 μL of FRAP reagent was mixed with 20 μL of the sample or 20 μL of various concentrations of Trolox standards (a synthetic water‐soluble analog of vitamin E) or 20 μL of distilled water (blank). The reaction mixture was incubated for 8 min at 37°C, and the absorbance was read at 600 nm using a Microplate Reader (BioTek® Instruments, Vermont, and USA). Pooled plasma from 10 apparently healthy individuals was used as a quality control with an interassay coefficient of variation (CV) of 1.5%. The concentration of antioxidants in the plasma was expressed as μM Trolox equivalents/mL (TE/mL) (Benzie & Strain, 1996).

### 2.7 Measurement of lymphocyte subpopulations

Total leukocytes and lymphocyte count were obtained from complete blood count analysis. The proportion and absolute number of lymphocyte subsets from whole blood were analyzed by flow cytometry using commercially available procedures (The BD Multitest IMK kit: BD Multitest™ CD3/CD8/CD45/CD4 reagent and BD Multitest™ CD3/CD16 + CD56/CD45/CD19 reagent). These antibodies are labeled as follows: PerCP‐anti‐CD45, FITC‐anti‐CD3, APC‐anti‐CD4, PE‐anti‐CD8, APC‐anti‐CD19, PE‐anti‐CD16, and PE‐anti‐CD56; all fluorescence antibodies were purchased from BD Biosciences (San Jose, CA, USA). Briefly, the whole blood 100 μL was incubated with 10 μL of BD Multitest™ reagent solution in the dark at room temperature for 15 min. The erythrocytes were lysed by FACS lysing solution. After 15 min of incubation, stained cells were analyzed by BD FACSCalibur™ (Becton Dickinson, San Jose, CA, USA). The percentages and absolute counts of human lymphocyte subsets: T cells: CD45 + CD3+, CD4 + T cells: CD45 + CD3 + CD4 + CD8− and CD8+ T cells: CD45 + CD3 + CD4 − CD8+ were analyzed using FlowJo software (Tree Star, Ashland, OR, USA).

### 2.8 Measurement of leukocyte telomere length

Peripheral blood mononuclear cells (PBMC) were isolated from whole blood by density gradient centrifugation using Ficoll‐Paque (Pharmacia Biotech, Uppsala, Sweden). Genomic DNA was extracted from 2 to 5 million PBMCs of study samples using a column‐based method with the DNAeasy kit (Qiagen, Germany) according to the manufacturer's protocol. The purity and quantity of the genomic DNA were determined using 260/280 ultraviolet spectrophotometer. PBMC telomere length was measured by a real‐time PCR‐based assay that compared telomere repeat sequence copy number to the single‐copy gene 36B4. Absolute telomere length (aTL) of DNA from PBMC was measured by determining the number of TTAGGG hexamer repeats using quantitative real‐time PCR (qPCR) (QuantStudio™ 5 Real‐Time PCR machine, Thermo Fisher, USA). The method was based on the qPCR technique described by O'Callaghan and Fenech (2011). To validate the assay, a 6‐point standard curve was run from a pool of control DNAs unrelated to the study for the telomere and 36B4 gene to ensure the linearity of the reaction (*R* ^2^ > 0.99). Briefly, each 10‐μL reaction was performed as follows; 20‐ng DNA template, 1xPowerUp SYBR Green Master Mix, 10 μM telomere forward primer, and 10‐μM telomere reverse primer. Cycling conditions were the initial holding stage at 95°C for 10 min, followed by 40 cycles of 95°C for 15 s and 60°C for 1 min., followed by a dissociation curve. The number of diploid genomes was determined using the 36B4 (a single copy gene) amplicon with the same cycle and condition as the telomere amplicon. The standard curve was used to convert relative telomere length values to aTL data which were reported as kb/diploid genome. Each standard concentration of standard DNA and samples was run as triplicates. The interassay coefficient of variation for telomere length measurement was 1% in this study. The oligomers used in this study are shown in Table S1.

### 2.9 Statistical analysis

#### 2.9.1 Sample size

A sample size of 20 subjects per group was estimated based on the mean ± SD difference in telomere length after 8 weeks of consuming the product (five capsules/day), which was found to be 408 ± 577.4. As mentioned in the introduction, these data were preliminary collected from 10 apparently healthy voluntary consumers who were willing to collect blood for measurement of telomere length. The estimation considered a desired power of 80%, a significance level of 0.05, and an anticipated dropout rate of 20%.

#### 2.9.2 Data analysis

The baseline characteristics of the participants were compared between groups using independent t‐test and chi‐square test, as appropriate. Pearson correlation was used to describe cross‐sectional associations between continuous biomarkers. A two‐way repeated measures ANOVA was used to analyze the main effects of the intervention over time, both within and between groups. A Bonferroni post hoc test was then used to calculate significant differences between groups and compare between times within group. The statistical analyses were performed using GraphPad Prism 6.0, with a significance level of 5% (*p* < 0.05) for all analyses.

## 3 RESULTS

**FIGURE 1:**
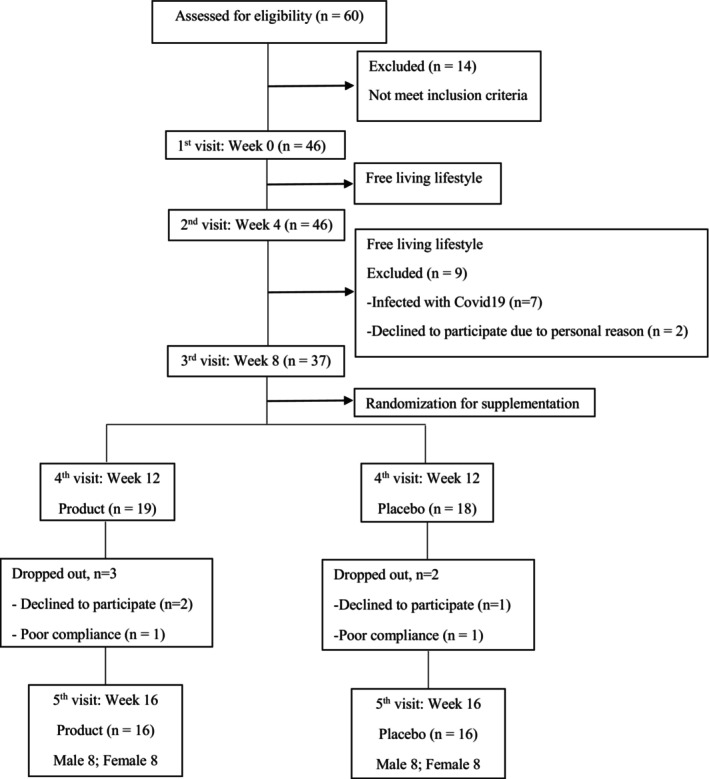
Study flowchart.

Figure 1 shows the CONSORT flow diagram of the study trial. A total of 60 applicants were screened for eligibility, out of which 14 did not meet the inclusion criteria. Eventually, 46 participants were enrolled and underwent the baseline assessment (first visit). During the 8 weeks of free‐living phase (second and third visit), nine participants were excluded, seven participants discontinued the intervention due to COVID‐19 infection, and two participants could not comply with the protocol due to job‐related issues. Consequently, 37 participants (fourth visit) were randomized for the 8‐week supplementation phase, with 19 and 18 participants allocated to receive product and placebo, respectively. During the 8‐week supplementation phase, two participants and three participants in the placebo and product group, respectively, had low compliance. Only 16 participants per group (eight males and eight females per group) completed the study. Mild intestinal constipation was reported in three cases in the placebo group.

### 3.1 Effect of product on general characteristics and blood biochemistry of participants

Thirty‐two participants completed the intervention trial, with 16 participants (eight males and eight females) in each group. The average age was 55.2 ± 3.2 years in the placebo group and 56.2 ± 4.4 years in the product group. At baseline, there were no significant differences in general characteristics and blood biochemistry between the placebo and product groups (Table 1). After the 16‐week study period (five visits), the blood biochemistry parameters did not differ significantly within or between the placebo and product groups. In particular, the levels of serum BUN, creatinine, AST, and ALT, which indicated liver and kidney function, were all within the normal range. Body weight, BMI, % body fat, and visceral fat did not change significantly within or between the two groups (Table S2). In addition, the proportion of dietary intake did not significantly differ within or between the two groups, except for protein intake during weeks 13–16, which was significantly higher in the placebo group compared to weeks 0–4 (Table S3).

**TABLE 1:** Blood biochemistry values during the study period in the intervention groups (mean ± SD).

Parameters	Placebo (*n* = 16)					Product (*n* = 16)				
	Free living			Placebo		Free living			Product	
	Baseline	Week 4	Week 8	Week 12	Week 16	Baseline	Week 4	Week 8	Week 12	Week 16
Hb, g/dL	13.6 ± 1.2	13.4 ± 1.1	13.4 ± 1.3	13.5 ± 1.1	13.6 ± 1.2	13.5 ± 1.2	13.4 ± 1.0	13.4 ± 1.2	13.6 ± 1.2	13.5 ± 1.1
FBS, mg/dL	93 ± 9	91 ± 8	91 ± 7	90 ± 7	90 ± 7	91 ± 7	89 ± 6	90 ± 5	90 ± 7	92 ± 7
Total cholesterol, mg/dL	228 ± 25	226 ± 27	229 ± 25	228 ± 26	234 ± 36	214 ± 33	217 ± 23	225 ± 24	223 ± 28	219 ± 21
HDL‐C, mg/dL	65 ± 13	65 ± 14	67 ± 14	67 ± 15	65 ± 16	66 ± 11	64 ± 12	65 ± 11	65 ± 12	65 ± 13
LDL‐C, mg/dL	142 ± 22	138 ± 27	138 ± 18	143 ± 21	143 ± 30	130 ± 30	132 ± 21	138 ± 22	137 ± 26	134 ± 20
Triglyceride, mg/dL	103 ± 36	103 ± 51	100 ± 40	92 ± 30	115 ± 53	92 ± 27	93 ± 46	97 ± 39	101 ± 53	94 ± 28
SGOT, U/L	23 ± 6	23 ± 7	22 ± 4	21 ± 5	22 ± 6	23 ± 6	22 ± 4	23 ± 4	23 ± 5	24 ± 8
SGPT, U/L	22 ± 10	20 ± 11	19 ± 8	20 ± 6	22 ± 9	19 ± 7	18 ± 6	19 ± 5	19 ± 6	23 ± 12
Uric acid, mg/dL	5.7 ± 1.3	5.8 ± 1.2	5.7 ± 1.3	5.6 ± 1.1	5.5 ± 1.1	5.6 ± 1.4	5.6 ± 1.4	5.6 ± 1.6	5.4 ± 1.4	5.8 ± 1.5
BUN, mg/dL	14 ± 3	14 ± 3	14 ± 4	14 ± 4	14 ± 4	14 ± 3	14 ± 3	13 ± 3	13 ± 3	14 ± 3
Creatinine, mg/dL	0.88 ± 0.2	0.90 ± 0.2	0.93 ± 0.2	0.94 ± 0.2	0.95 ± 0.2	0.92 ± 0.2	0.92 ± 0.2	0.92 ± 0.2	0.93 ± 0.2	1.00 ± 0.2

Abbreviations: BUN, Blood urea nitrogen; FBS, Fasting blood sugar; Hb, Hemoglobin; HDL‐C, High‐density lipoprotein cholesterol; LDL‐C, Low‐density lipoprotein cholesterol; SGOT, Serum glutamic oxaloacetic transaminase; SGPT, Serum glutamate‐pyruvate transaminase.

### 3.2 Effect of product supplementation on leukocyte telomere length

As shown in Table 2, there were no differences in absolute and percentile of telomere length between the placebo and product groups (6629 ± 1116 bp; p53 ± 21 and 6313 ± 1204 bp; p47 ± 24, respectively) at baseline. Additionally, both the absolute and percentile telomere length did not significantly change during the free‐living phase (baseline, week 4, and week 8) both within and between groups. After 8 weeks of supplementation, the absolute telomere length was significantly increased from 6313 ± 1204 bp at baseline to 6655 ± 1357 bp at week 16 in the product group (*p* < 0.05), while the percentile was significantly increased from 47 at baseline to 54 at week 16 (*p* < 0.05). In addition, the absolute telomere length at week 16 was significantly longer than that of week 12 (*p* < 0.05). In the placebo group, there were no significant changes in telomere length throughout the study.

**TABLE 2:** Telomere length, leukocyte count, lymphocyte subpopulation, and plasma total antioxidant capacity values during the study period in the intervention groups (mean ± SD).

Parameters	Placebo (*n* = 16)					Supplement (*n* = 16)				
	Free living			Placebo		Free living			Product	
	Baseline	Week 4	Week 8	Week 12	Week 16	Baseline	Week 4	Week 8	Week 12	Week 16
Telomere, base pairs	6629 ± 1116	6537 ± 1149	6650 ± 1020	6641 ± 1075	6724 ± 1121	6313 ± 1204	6312 ± 1107	6457 ± 1227	6522 ± 1380	6655 ± 1357^a^ ^,^ ^b^
Telomere, percentile	53 ± 21	51 ± 2	54 ± 17	54 ± 19	55 ± 21	47 ± 24	47 ± 22	50 ± 24	51 ± 26	54 ± 26^a^
WBC, cells/μL	5913 ± 1045	6164 ± 319	5998 ± 1013	5984 ± 1187	5822 ± 980	6122 ± 1398	6304 ± 1293	6305 ± 1353	6213 ± 1424	6349 ± 1400
Lymphocytes, cells/μL	2108 ± 485	2228 ± 644	2259 ± 414	2205 ± 495	2128 ± 403	2279 ± 493	2332 ± 507	2320 ± 527	2325 ± 474	2401 ± 705
CD 4, cells/μL	728 ± 182	763 ± 221	759 ± 181	744 ± 177	740 ± 176	789 ± 245	772 ± 249	790 ± 253	776 ± 256	776 ± 262
CD 4, %	35 ± 7	35 ± 7	34 ± 7	34 ± 7	35 ± 6	35 ± 7	33 ± 7	34 ± 7	33 ± 7	33 ± 7
CD 8, cells/μL	557 ± 208	592 ± 281	597 ± 195	573 ± 208	560 ± 205	594 ± 193	618 ± 216	621 ± 224	608 ± 219	649 ± 267
CD 8, %	26 ± 6	26 ± 6	26 ± 7	26 ± 6	26 ± 7	26 ± 6	27 ± 7	27 ± 6	26 ± 6	27 ± 6
CD4: CD 8	1.37 ± 0.32	1.40 ± 0.39	1.34 ± 0.35	1.37 ± 0.32	1.42 ± 0.39	1.41 ± 0.47	1.34 ± 0.52	1.39 ± 0.52	1.36 ± 0.47	1.31 ± 0.43
TAC, μM TE/mL^c^	999 ± 218	1010 ± 199	1001 ± 221	962 ± 187	966 ± 174	949 ± 164	937 ± 192	945 ± 191	909 ± 191	978 ± 207

Abbreviations: CD, Cluster of differentiation; TAC, total antioxidant capacity; WBC, White blood cell count.

^a^ Significant difference from baseline of each group at *p* < 0.05.

^b^ Significant difference from week 12 of each group, *p* < 0.05.

^c^ Plasma total antioxidant capacity by ferric ion reducing antioxidant power (Trolox equivalent/milliliter).

### 3.3 Effect of product supplementation on total antioxidant capacity (TAC)

**FIGURE 2:**
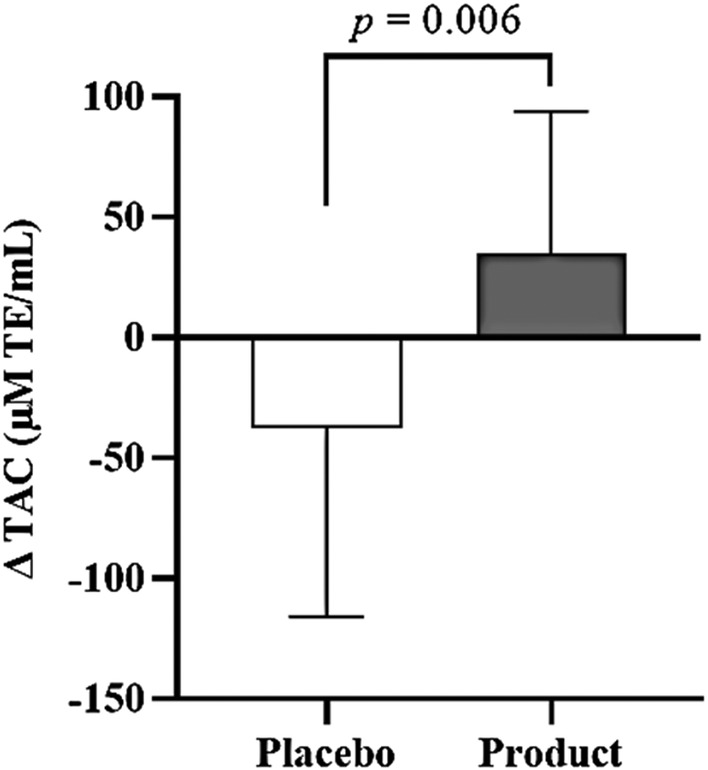
Mean of ∆ TAC between the placebo and product groups. Solid box and error bar represent the mean of ∆ TAC and SD. There is a significant difference between the placebo and product groups (*p*‐value = 0.006, *n* = 16). ∆ TAC represents the change in the total antioxidant capacity between the value at week 16th and the average value during the free‐living phase.

There were no significant differences in plasma total antioxidant capacity level between the placebo (999 ± 218 μM TE/mL) and product groups (949 ± 164 μM TE/mL) at baseline (Table 2). The absolute plasma total antioxidant capacity did not show significant differences within and between the groups throughout the study period. However, the mean change between TAC at week 16 and average TAC during the free‐living phase increased by 35 ± 59 μM TE/mL in the product group compared to a decrease of −38 ± 78 μM TE/mL in the placebo group, and the difference in TAC change between the groups was statistically significant (*p* = 0.006) (Figure 2).

### 3.4 Effect of product supplementation on leukocyte count and lymphocyte subset

There were no significant differences in total leukocyte count, total lymphocytes, total CD4+ and CD8+, % CD4+, and % CD8+ T cells between the placebo and product groups at baseline (Table 2). These blood parameters were within normal ranges throughout the study period, and no significant differences were observed for these blood cell parameters both within and between groups (Table 2).

### 3.5 Association between telomere length with the proportion of CD4+ and CD8+ and visceral fat

**FIGURE 3:**
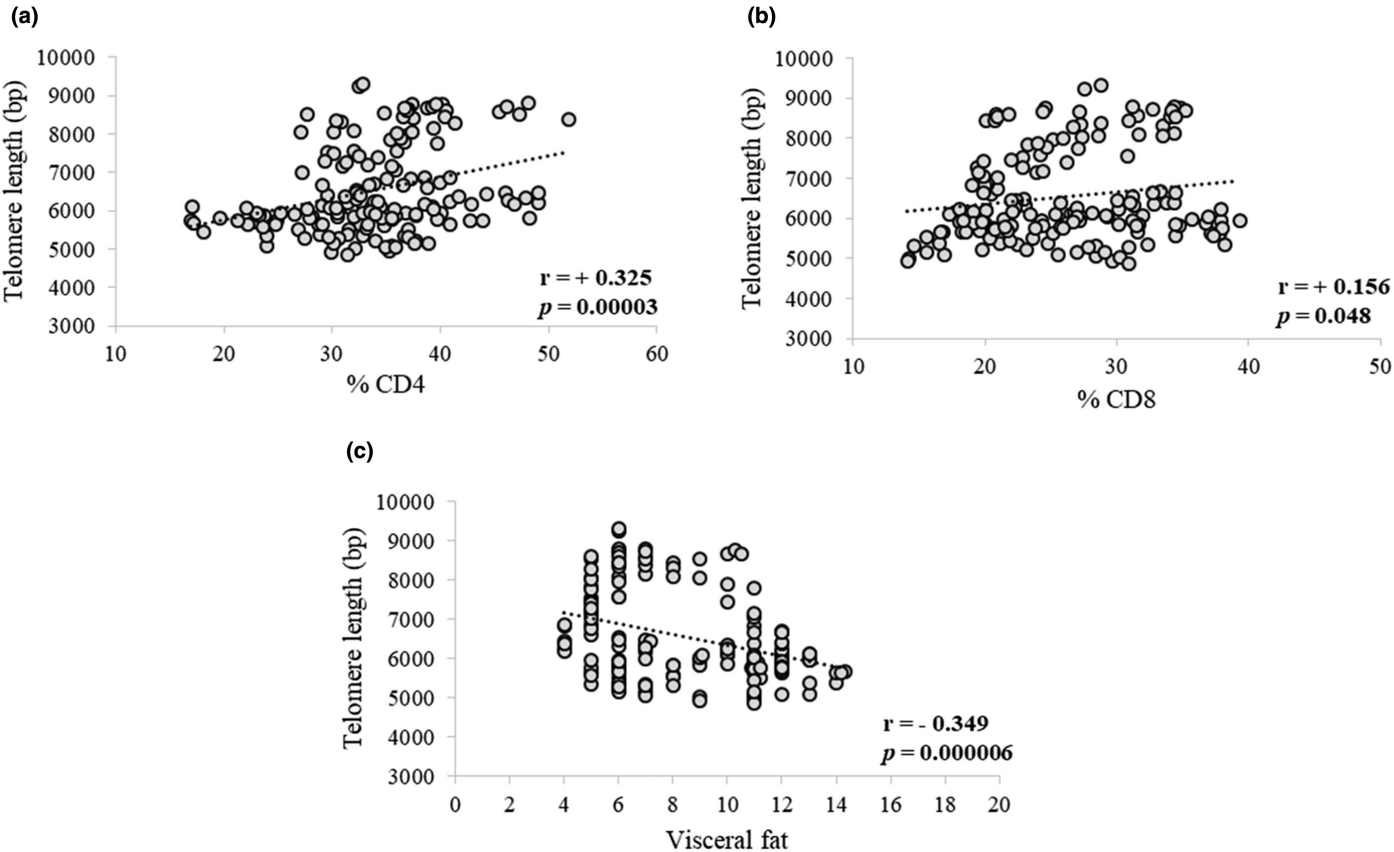
Correlation analysis between telomere length and % CD4 (a), % CD8 (b), and visceral fat (c) (*n* = 160). The data were evaluated using the Pearson correlation coefficient (*r*).

Although no significant differences were observed in total leukocytes, total lymphocytes, total CD4+, CD8+, % CD4+, and % CD8+ both within and between the placebo and product groups throughout the study period, a positive correlation was found between telomere length and % CD4+ at *r* = +0.325 (*p* < 0.0001) and % CD8+ at *r* = +0.156 (*p* = 0.048), as shown in Figure 3a,b, respectively. In addition, a negative correlation between telomere length and visceral fat was observed [*r* = −0.349 (*p* < 0.0001)] as shown in Figure 3c.

## 4 DISCUSSION

Previous studies have shown that telomeres could either shorten or elongate in vivo (Shlush et al., 2011; Svenson et al., 2011). Lengthening of telomere length can be achieved through activation of cellular telomerase activity or reduction in exposure to factors that induce telomere attrition. The present study demonstrated that 8 weeks of supplementation with five capsules of product significantly increased leukocyte telomere length while no significant difference was observed in the placebo group throughout the study. However, a large number of sample size needs to be conducted in the future to explore additional mechanisms of this product on telomere length. This positive effect on telomere length might mediate through the positive change in plasma total antioxidant capacity in the product group (Figure 2). However, product supplementation did not significantly influence the number of leukocytes, total lymphocytes, and T‐lymphocyte subsets.

Oxidative stress is highlighted to be the main factor that accelerates telomere shortening (von Zglinicki, 2002). An imbalance between antioxidant and free radical production in human body leads to induced oxidative stress status. Oxidative damage slowly increases over time within the body due to the physiological decline of antioxidant defenses that occurs during aging (Osterod et al., 2001). A review has reported a positive correlation between telomere length and antioxidant status in humans (Reichert & Stier, 2017). The antioxidant activity of five edible plants in the product might play a role in lengthening the leukocyte telomere length in the present study.

Pennywort and its bioactive compounds, asiaticoside and madecassoside, have demonstrated a wide range of therapeutic potential, including antioxidant and anti‐inflammatory effects in various organs (Bandopadhyay et al., 2023; Sun et al., 2020). A recent study found that the water extract of pennywort leaves could activate the nuclear factor erythroid 2‐related factor 2 (NRF2), which regulates the antioxidant response pathway in aged mice (Zweig et al., 2021). Black sesame seeds also possess antioxidative effects, as shown in a previous study where a 4‐week administration of black sesame meal significantly decreased malondialdehyde (MDA) levels and increased vitamin E levels (Wichitsranoi et al., 2011). Another study showed that sesame consumption reduced oxidative stress by significantly reducing MDA levels but increasing superoxide dismutase (SOD) in soccer players (Barbosa et al., 2017). Guava fruits are also rich in antioxidant agents (Naseer et al., 2018). A study in 28 young men found that 4 weeks of consumption of guava fruits significantly increased plasma total antioxidant capacity compared to baseline (*p* < 0.05) (Rahmat et al., 2006). *Garcinia mangostana*, or mangosteen fruits, are a good source of nutrients and bioactive compounds such as xanthones, benzophenones, hydroxycitric acid, and anthocyanins, which possess antioxidant, anti‐inflammatory, anticancer, antimicrobial, antiallergy, antiulcer, antiparasitic, and antihelminthic activities (Murthy et al., 2019). A human study reported that daily consumption of a mangosteen product drink for 30 days increased 15% more antioxidant capacity than placebo in healthy adults and decreased C‐reactive protein 46% relative to the baseline value (Xie et al., 2015). Soybeans contain several bioactive compounds, including isoflavones, saponins, phytic acids, phytosterols, trypsin inhibitors, and peptides, which have positive impacts on human health (Isanga & Zhang, 2008). Soybean isoflavones were reported to protect DNA oxidation in human lymphocytes (Mitchell & Collins, 1999). Administration of dietary genistein (250 ppm/kg diet) also enhanced antioxidant enzyme activities in various mouse organs, including the small intestine, liver, and kidney (Cai & Wei, 1996). Another study found that diabetic rats fed with isolated soy protein‐based diet (containing isoflavone 189 mg/100 g isolated soy protein) for 3 weeks had a greater heart antioxidant enzyme activity (SOD, catalase, and glutathione S‐transferase) than control rats fed with casein‐based diet (Mendes et al., 2014). Consumption of soy protein rich in isoflavones has been shown to reduce the susceptibility of LDL oxidation in healthy subjects (Jenkins et al., 2002, 2003). Soybean‐derived bioactive peptides exhibit multiple functional properties in humans, such as antioxidative, antithrombotic, antimicrobial, immunoregulatory, opiate‐like, mineral‐binding, hypocholesterolemic, and antihypertensive effects (Kim et al., 2021). An experimental study found that rats supplemented with 3.333 g/kg body weight of soy protein for 63 days decreased plasma MDA content but significantly increased plasma total antioxidant capacity and liver glutathione peroxidase compared with those of the control group (Ali et al., 2015). Another human study reported that soy protein consumption could preserve plasma antioxidant capacity in exercise training lean body mass males (Brown et al., 2004).

Although telomerase activity was not measured in the present study, telomere length was used as an indirect parameter for prediction of telomerase activity. Isoflavones in soybean have estrogen‐like activities or weak estrogen, which are proposed as an alternative hormone replacement therapy for postmenopausal women (Cassidy et al., 1994). Estrogen has been demonstrated to upregulate telomerase reverse transcriptase (TERT) gene expression and telomerase activity in estrogen‐deficient mice after 3 weeks of supplementation with estrogen, which was able to restore TERT gene expression and telomerase activity (Bayne et al., 2008). The soybean extract has been shown to have telomerase activator effect in diabetes‐induced rats. Supplementation with soybean extract increased the amount of TERT and restored telomerase function resulting in an increase in the number of pancreatic β‐cells in diabetes‐induced rats (Mustofa et al., 2019). Additionally, pennywort leaves have demonstrated telomerase activator activity. An in vitro study showed that the 08AGTL formulation from pennywort extract could trigger a 9‐fold increase in telomerase activity compared to the untreated human PBMC (Tsoukalas et al., 2019). Another study reported that the DLBS1649 formulation from pennywort extract could repress telomere shortening by 25% relative to the untreated age human kidney cell line (HEK293 cells) by repressing the decrease in telomerase protein expression by 18% relative to the untreated age HEK293 cells (Karsono et al., 2021). Recently, *C. asiatica* leaf (CA) extract and its bioactive compound, madecassoside (MD), were demonstrated to activate telomerase in human colonic organoids after 1 week of treatment. Consistently, the telomere length of ulcerative colitis‐model organoids was significantly improved by a 6‐week treatment with CA or MD (Watanabe et al., 2022).

The present study found a negative association between telomere length and visceral fat. This finding is consistent with data from a previous study that also found a negative association between relative telomere length and the percentage of body fat and visceral fat (Nantanawut et al., 2022). Furthermore, other supportive studies have shown a clear negative correlation between obesity and telomere length (Khosravaniardakani et al., 2022; Nonino et al., 2018). Obesity is a vital underlying factor in the development of metabolic syndrome. Fat accumulation in obesity has been shown to have a positive correlation with systemic oxidative stress in both humans and mice. The increased oxidative stress associated with accumulated fat is an important pathogenic mechanism of obesity‐associated metabolic syndrome. In adipose tissues of obese mice, reactive oxygen species (ROS) levels have been shown to increase, while the expression of antioxidative enzymes decreased. Treatment with NADPH oxidase inhibitor has been found to reduce ROS production in adipose tissues (Furukawa et al., 2004). ROS can damage DNA bases, with guanine being particularly susceptible due to its low redox potential. Telomeres are composed of G‐rich TTAGGG repeats, which makes them highly sensitive to oxidative stress damage, as they contain a high number of guanines with low redox potential (Poli et al., 2004). A previous study showed a positive correlation between weight loss and telomere lengthening, with the most significant telomere lengthening observed in participants with the shortest telomeres at baseline (Carulli et al., 2016).

The present study found a positive correlation between PBMC telomere length and the percentage of CD4+ and CD8+ T cells. These positive correlations may link to the capability of T cells to eliminate infections. A recent study on the aging population found that a low number of CD4+ and CD8+ T lymphocytes contributed to a poor prognosis in severe cases of COVID‐19 (Tan et al., 2020). This may be a consequence of replicative failure of immune cells due to early lymphocyte senescence. A decline in immune function with age has been shown in a larger cohort, where telomerase activity declines with age in resting T cells and activated T cells (Lin et al., 2015). Thus, it is not surprising that longer leukocyte telomeres are associated with better survival from sepsis and lower severity of acute respiratory syndrome in critically ill patients (Liu et al., 2020).

The present study has three limitations that need to be considered. Firstly, the changes in telomerase activity were not evaluated. The change in telomerase activity should be assessed in future studies to define the mechanism of the product in delaying telomere attrition or lengthening telomere length after product consumption. Secondly, oxidative stress biomarkers in particular DNA‐damaged biomarkers (such as 8‐oxodeoxyguanine and 2′‐deoxy‐7, 8‐dihydro‐2′‐deoxyadenosine), which are associated with telomere attrition should be measured. Additionally, plasma enzymatic and nonenzymatic antioxidant agents should be assessed after product consumption. These would provide additional information to understand the action mechanisms of the product on telomere length. Lastly, the content of functional bioactive compounds of each plant in the product should be further identified because the amount of such functional bioactive compounds reflects the functional efficacy of the product. However, the content of functional active ingredients in the products will be analyzed in the future research.

One of the strengths of the present study was the inclusion of an 8‐week free‐living phase for all biomarkers, particularly telomere length, before starting the supplementation phase. This ensured that all participants had a stable metabolism by maintaining their habitual lifestyle in terms of dietary intake, exercise, or medication. All participants had similar intakes of macronutrients and energy, throughout the supplementation phase. The lack of significant differences between groups confirms that the daily dietary intake did not have any influence on the outcomes in this study. Furthermore, only menopausal female participants were recruited to rule out any potential positive effect of estrogen on telomere length.

Regarding the safety issue, daily consumption of five capsules/day meant that individual consumed pennywort leaf powder 750 mg (equivalent to 3.75 g fresh weight); black sesame seed powder 2 g (equivalent to10 g fresh weight); isolated soy protein powder 400 mg (equivalent to 2 g fresh weight); guava fruit juice powder 2 g (equivalent to 10 g fresh weight), and mangosteen aril juice powder 250 mg (equivalent to 1.25 g fresh weight). This supplementation dose should not generate adverse effects as documented in the previous studies. A previous study reported no significant elevation in the levels of liver enzymes after a 6‐week treatment with 750 mg or 1000 mg of pennywort leaf extract compared to their baseline levels (Farhana et al., 2016). Another study reported that the consumption of 20 g/day of soy protein isolate for 2 years was well tolerated and safe (Bosland et al., 2013). Additionally, healthy subjects supplemented with 400 g of guava fruit for 6 weeks did not show any observable adverse effects (Kumari et al., 2016). Furthermore, participants with an average age of 55 years supplemented with 1000 mg of mangosteen fruit pulp powder for 28 days did not report any adverse effects (Rodriguez et al., 2014). Lastly, no adverse events were observed in elderly individuals aged 60–80 years who consumed 20 g/day of black sesame seeds for 12 weeks (Saisum et al., 2020). In the present study, no product‐related toxicities were observed after 8 weeks of supplementation indicating by the blood biochemistry markers of liver, kidney, and other metabolic functions, which did not show any abnormal findings.

## 5 CONCLUSIONS

Daily consumption of five capsules of Mylife/Mylife100® for 8 weeks was safe and increased leukocyte telomere length probably by improving plasma total antioxidant capacity, without any changes of the other variables. Mylife/Mylife100® consumption might be an alternative approach for slowing biological aging and might prevent chronic diseases associated with oxidative stress. However, a larger number of sample size needs to be conducted in the future to define additional mechanisms of these supplements on telomere length.

## AUTHOR CONTRIBUTIONS

**Kemika Praengam:** Data curation (equal); formal analysis (equal); investigation (equal); methodology (equal); validation (equal); writing – original draft (supporting). **siriporn Tuntipopipat:** Conceptualization (lead); data curation (lead); formal analysis (lead); funding acquisition (lead); investigation (lead); methodology (equal); validation (lead); writing – original draft (lead); writing – review and editing (lead). **Chawanphat Muangnoi:** Conceptualization (supporting); data curation (equal); formal analysis (equal); investigation (equal); methodology (equal); validation (equal); writing – original draft (supporting). **Chatdao Jangwangkorn:** Conceptualization (supporting); investigation (supporting); writing – review and editing (supporting). **Olan Piamkulvanich:** Conceptualization (supporting); methodology (equal); validation (equal).

## FUNDING INFORMATION

This research was funded by Asian Phytoceuticals Public Company Limited which is the only funding sponsor without influence on the study trial.

## CONFLICT OF INTEREST STATEMENT

Asian Phytoceuticals Public Company Limited is the only funding sponsor without influence on the study trial.

## Supporting information


Table S1

Table S2

Table S3

## Data Availability

Not applicable.
