# Supplementary material for: Efficacy of a dietary supplement derived from five edible plants on telomere length in Thai adults: A randomized, double‐blind, placebo‐controlled trial
Source: Food Sci Nutr. 2023 Nov 20;12(3):1592–604. doi: 10.1002/fsn3.3851 (PMC10916585; doi:10.1002/fsn3.3851)
Supplement: Supplementary file 1 — Table S1 [file FSN3-12-1592-s002.docx]

**Supplementary Table 1.** Oligomers used for aTL assay

|  | Oligomer name | Sequence 5´- 3´ |
| --- | --- | --- |
| Standards | Telomere standard | (TTAGGG)14 |
|  | 36B4 standard | CAGCAAGTGGGAAGGTGTAATCCGTCTCCACAGACA  AGGCCAGGACTCGTTTGTACCCGTTGATGATAGAATGGG |
| PCR primers | teloF | CGGTTTGTTTGGGTTTGGGTTTGGGTTTGGG TTTGGGTT |
|  | teloR | GGCTTGCCTTACCCTTACCCTTACCC TTACCCTTACCCT |
|  | 36B4F | CAGCAAGTGGGAAGGTGTAATCC |
|  | 36B4R | CCCATTCTATCATCAACGGGTACAA |
