# Supplementary material for: Efficacy of a dietary supplement derived from five edible plants on telomere length in Thai adults: A randomized, double‐blind, placebo‐controlled trial
Source: Food Sci Nutr. 2023 Nov 20;12(3):1592–604. doi: 10.1002/fsn3.3851 (PMC10916585; doi:10.1002/fsn3.3851)
Supplement: Supplementary file 2 — Table S2 [file FSN3-12-1592-s003.docx]

**Supplementary Table 2.** Body composition of participants throughout the study period (mean±SD)

| **Parameters** | **Placebo** | | | | | **Product** | | | | |
| --- | --- | --- | --- | --- | --- | --- | --- | --- | --- | --- |
|  | **Free Living** | | | **Placebo** | | **Free Living** | | | **Product** | |
|  | **Baseline** | **Week 4** | **Week 8** | **Week 12** | **Week 16** | **Baseline** | **Week 4** | **Week 8** | **Week 12** | **Week 16** |
| **BW, kg** | 60.7±7.5 | 60.6±7.6 | 60.6±7.6 | 60.7±7.7 | 60.9±7.6 | 58.3±8.8 | 57.8±8.8 | 57.8±9.2 | 57.5±9.1 | 57.4±9.3 |
| **BMI, kg/m^2^** | 22.7±1.4 | 22.6±1.4 | 22.6±1.4 | 22.7±1.4 | 22.7±1.4 | 22.4±1.6 | 22.2±1.5 | 22.2±1.7 | 22.0±1.7 | 22.0±1.7 |
| **Body fat, (%bw)** | 25±6 | 26±6 | 26±6 | 26±6 | 24±9 | 25±7 | 25±7 | 25±7 | 25±7 | 25±7 |
| **Visceral fat** | 8.7±3 | 8.6±3 | 8.1±3 | 8.0±3 | 7.9±4 | 8.4±3 | 8.2±3 | 8.2±3 | 7.9±3 | 8.0±3 |

*BW; Body Weight, BMI; Body mass index*
