# Supplementary material for: Efficacy of a dietary supplement derived from five edible plants on telomere length in Thai adults: A randomized, double‐blind, placebo‐controlled trial
Source: Food Sci Nutr. 2023 Nov 20;12(3):1592–604. doi: 10.1002/fsn3.3851 (PMC10916585; doi:10.1002/fsn3.3851)
Supplement: Supplementary file 3 — Table S3 [file FSN3-12-1592-s001.docx]

**Supplementary Table 3.** Energy requirement, energy intake, and energy distribution throughout the study (mean±SD)

| **Parameters** | **Placebo (n=16)** | | | | **Product (n=16)** | | | |
| --- | --- | --- | --- | --- | --- | --- | --- | --- |
|  | **Wk0-4** | **Wk5-8** | **Wk9-12** | **Wk13-16** | **Wk0-4** | **Wk5-8** | **Wk9-12** | **Wk13-16** |
| Energy requirement, kcal | 1524±220 | 1520±207 | 1524±217 | 1530±212 | 1466±295 | 1470±301 | 1443±298 | 1442±294 |
| Energy intake, kcal | 1566±403 | 1468±402 | 1459±307 | 1410±199 | 1355±307 | 1430±297 | 1365±249 | 1368±237 |
| % of energy requirement | 102±18 | 96±20 | 96±14 | 93±13 | 93 ±15 | 98±15 | 97±22 | 97±19 |
| **Energy distribution, % of total energy** | | | | | | | | |
| Carbohydrate | 54.8±6.5 | 53.8±6.7 | 52.5±6.3 | 51.9±4.2 | 51.8±6.7 | 51.4±8.0 | 50.8±8.5 | 50.1±9.8 |
| Protein | 14.8±1.8 | 15.6±2.7 | 16.1±2.7 | 16.1±1.9^*^ | 17.0±4.3 | 16.2±2.8 | 16.9±3.9 | 16.9±3.4 |
| Fat | 30.4±5.5 | 30.8±5.5 | 31.5±4.9 | 32.0±3.7 | 31.2±4.6 | 32.4±6.2 | 32.8±6.3 | 33.3±6.0 |
| Dietary Fiber, g | 10.6±2.9 | 10.8±2.9 | 10.0±3.2 | 10.1±2.9 | 8.9±2.0 | 8.9±1.9 | 8.3±2.2 | 8.8±3.0 |
| Cholesterol, mg | 240±125 | 228±130 | 213±101 | 222±77 | 250±121 | 259±93 | 232±111 | 218±80 |

^*^ Significant difference from week 0-4 of each group
